# Biventricular function in preterm infants with patent ductus arteriosus ligation: A three-dimensional echocardiographic study

**DOI:** 10.1038/s41390-024-03180-w

**Published:** 2024-04-13

**Authors:** Katsuaki Toyoshima, Hirosato Aoki, Takahiro Noguchi, Naka Saito, Tatsuto Shimizu, Takahiro Kemmotsu, Tomoyuki Shimokaze, Tomoko Saito, Jun Shibasaki, Motoyoshi Kawataki, Toshihide Asou, Tsuyoshi Tachibana, Satoshi Masutani

**Affiliations:** 1 Department of Neonatology, Kanagawa Children’s Medical Center, Yokohama, Japan; 2 Department of Clinical Laboratory, Kanagawa Children’s Medical Center, Yokohama, Japan; 3 Department of Cardiovascular Surgery, Kanagawa Children’s Medical Center, Yokohama, Japan; 4 Department of Pediatrics, Saitama Medical Center, Saitama Medical University, Kawagoe, Japan

## Abstract

**Background:**

The detailed hemodynamics after patent ductus arteriosus (PDA) ligation in preterm infants remain unknown. We aimed to clarify the effect of surgical ligation on left ventricular (LV) and right ventricular (RV) volume and function.

**Methods:**

Echocardiography was performed in 41 preterm infants (median gestational age: 25 weeks) before and after PDA ligation. Global longitudinal strain was determined using three-dimensional speckle-tracking echocardiography. These values were compared with those in 36 preterm infants without PDA (non-PDA).

**Results:**

Preoperatively, the PDA group had greater end-diastolic volume (EDV) and cardiac output (CO) in both ventricles, a higher LV ejection fraction (LVEF) (53% vs 44%) and LV global longitudinal strain, and a lower RVEF (47% vs 52%) than the non-PDA group. At 4–8 h postoperatively, the two groups had a similar LVEDV and RVEDV. However, the PDA group had a lower EF and CO in both ventricles than the non-PDA group. At 24–48 h postoperatively, the RVEF was increased, but the LVEF remained decreased, and LVCO was increased.

**Conclusions:**

PDA induces biventricular loading and functional abnormalities in preterm infants, and they dramatically change after surgery. Three-dimensional echocardiography may be beneficial to understand the status of both ventricles.

**Impact:**

- Preterm infants are at high risk of hemodynamic compromise following a sudden change in loading conditions after PDA ligation.
- Three-dimensional echocardiography enables quantitative and serial evaluation of ventricular function and volume in preterm infants with PDA.
- PDA induces biventricular loading and functional abnormalities in preterm infants, and they dramatically change after surgery.

## Introduction

Preterm infants are at high risk of hemodynamic compromise following patent ductus arteriosus (PDA) ligation.^1^ Regardless of whether surgical or through a percutaneous approach, definitive closure of PDA leads to a sudden change in loading conditions of the left ventricle (LV). These conditions are associated with physiological abnormalities and lead to hypotension, low cardiac output (CO), and impairment of oxygenation and ventilation.^2–4^ Approximately 10–45% of preterm infants with surgical ligation of the PDA have post-ligation hemodynamic instability, which may affect the long-term outcome of this vulnerable infantile population.^5^

However, how hemodynamic instability occurs after the surgical closure of PDA remains unclarified. Several conventional echocardiographic studies have reported the changes in LV function associated with increased LV afterload following PDA closure.^3,4,6–10^ There are several limitations to the use of conventional echocardiography to evaluate LV volume and function. The estimation of LV volume and the ejection fraction (EF) through conventional echocardiography is based on linear geometrical assumptions that may not precisely represent true three-dimensional (3D) volume.^11–13^ In particular, M-mode estimates of LV volume and the EF are angle-dependent and may not be accurate because LV is not always round owing to transitional physiology, suggesting progressively decreasing pulmonary vascular resistance in the early neonatal period.

Right ventricular (RV) function is also important not only in those with pulmonary hypertension, but also in those with severely impaired LV function.^14,15^ We speculate that RV function plays an important role in preterm infants with PDA. RV function in this population has rarely been investigated. The LV has a conical structure with mitral-aortic fibrous continuity, while the RV has complex geometry with a crescent shape and non-continuity between the tricuspid and pulmonary valves. Evaluating RV volume and the EF is much more difficult with conventional two-dimensional (2D) echocardiography than evaluating LV volume and function. Recently, three-dimensional (3D) echocardiography has been used to determine LV and RV volumes and the EF without making geometric assumptions, and it is independent of the angle.^16,17^ Although careful attention needs to be paid to accurately obtain the volumes from the apex to avoid foreshortening, 3D echocardiography has high accuracy and reproducibility, similar to cardiovascular magnetic resonance.^18–23^ Although previous studies have shown that 3D echocardiography can quantify LV and RV volume and the EF in newborn infants,^24–26^ these parameters have not been fully investigated in preterm infants. This lack of investigation may be related to the limited validation of 3D echocardiography in preterm infants.

Current study aimed to test the hypothesis that not only LV, but also RV, function is acutely impaired after surgical PDA closure in preterm infants as shown by 3D echocardiography.

## Methods

### Study design and population

We performed a single-center, retrospective study. We included preterm infants who underwent PDA ligation with a gestational age of 23–33 weeks who were admitted to the neonatal intensive care unit between January 2017 and December 2022. We excluded infants with (1) cardiac anomalies other than patent foramen ovale (PFO) and persistent left superior vena cava, (2) multiple abnormalities or apparent clinical syndrome, and (3) chromosomal abnormalities.

The referrals for PDA ligation were triaged according to the clinical and echocardiographic findings. The following factors were indications for surgical closure of PDA: (1) further ventilatory support; (2) progressive congestive heart failure despite medical management or where cyclooxygenase inhibitors were contraindicated; (3) a transductal diameter > 1.5 mm, predominant left-to-right flow; (4) left atrial (LA) enlargement indicated by an LA/aortic diameter ratio (LA/Ao) > 1.3 or LA volume index > 1.0 ml/kg; and (5) left pulmonary artery end-diastolic velocity (LPA EDV) > 15 cm/s on echocardiography. Three-dimensional cardiac volume acquisition has been part of our standard protocol since 2017.

The indication for PDA surgery was determined by neonatologists who were unaware of the study design and were blinded to the 3D echocardiographic data. The LV and RV loading conditions and function before and after PDA ligation were evaluated by transthoracic echocardiography within 12 h before PDA ligation, within 4–8 h after PDA ligation, and between 24 and 48 h postoperatively. A power calculation was not performed because of the paucity of 3D echocardiographic data in this population.

LV and RV cardiac function in preterm infants with PDA (PDA group) before ligation was compared with that in those without PDA (non-PDA group). The inclusion criteria of the non-PDA group were as follows: (1) 14-day-old neonates with a gestational age between 23 and 28 weeks and those with a gestational age between 29 and 31weeks who required mechanical ventilation who were admitted to the neonatal intensive care unit between October 2020 and December 2022; (2) neonates who did not have PDA ligation performed; (3) PDA closure at day 14; and (4) neonates who did not have any congenital heart defects or pulmonary hypertension as indicated by tricuspid regurgitation pressure gradient >32 mmHg^27^ and/or a non-circular LV shape at the peak of systole. Three-dimensional cardiac volume acquisition for 14-day-old neonates with a gestational age between 23 and 28 weeks and those with a gestational age between 29 and 31weeks who required mechanical ventilation has been performed since 2020. The echocardiography dataset including 3D was acquired in all neonates with these criteria.

The preterm infants were treated according to our institutional protocols. The study was conducted in accordance with the principles contained in the Declaration of Helsinki and was approved by the institutional review board of Kanagawa Children’s Medical Center (No. 1806-07).

### Clinical characteristics

Data on sex, gestational age in weeks, birth weight, Apgar scores, the age (days) at PDA surgery, corrected gestational age in weeks, and body weight (BW) at the surgery day were collected from medical records. In the PDA group, treatment details, and survival or death at discharge, and additional characteristics were obtained.

All data, including baseline hemodynamics, respiratory characteristics, and echocardiograms, were obtained at the three time points mentioned above.

N-terminal pro-brain natriuretic peptide (NT-proBNP) concentrations were measured at pre-ligation and 24–48 h after surgery.

The clinical data collected included heart rate, blood pressure, oxygen saturation, the fraction of inspired oxygen, and mean airway pressure. Blood pressure was measured immediately before the echocardiography was performed. Postoperative blood pressure was measured using an arterial line at all time points in the PDA group. Blood pressure was measured with the oscillometer technique in all preterm infants in the non-PDA group and at some preoperative points in those in the PDA group without an arterial line.

At the time of the echocardiography, the respiratory severity score was calculated as the mean airway pressure (mmHg) × the fraction of inspired oxygen.^28^ The vasoactive–inotropic score at the initial echocardiographic examination was calculated as the dopamine dose (μg/kg/min) + the dobutamine dose (μg/kg/min) + 100 × the epinephrine dose (μg/kg/min) + 100 × the norepinephrine dose (μg/kg/min) + 10,000 × the vasopressin dose (U/kg/min) + 10 × the olprinone dose (μg/kg/min).^29^

### Three-dimensional echocardiography

Echocardiographic examinations were performed by an experienced echocardiographer (K Toyoshima). LV and RV function was evaluated using 3D echocardiography as part of our institutional protocol. Full-volume 3D datasets were acquired by the apical approach either from left-sided chest wall or subcostal using an ultrasound machine and equipment (EPIC 7 G or EPIC CVx with the X7-2 probe; Philips Healthcare, Andover, MA). The depth and sector angle were manipulated to include the entire LV or RV with a frame rate of > 40 frames/s. A full-volume scan was acquired from six R wave-triggered subvolumes to include the complete LV or RV into the 3D dataset. Six cardiac cycles in each capture were stitched together. We extracted 3D data over 6 cardiac cycles under a well-sedated level, no body motion, and no change in loading conditions.

The 3D echocardiography datasets for LV and RV were analyzed by an experienced investigator (K Toyoshima) using novel 3D echocardiography software (4D LV-Analysis version 3, 4D RV-Function version 3; TomTec Imaging Systems, Unterschleissheim, Germany). The accuracy and reproducibility for those have been validated by comparison with cardiovascular magnetic resonance.^30–32^

**Fig. 1:**
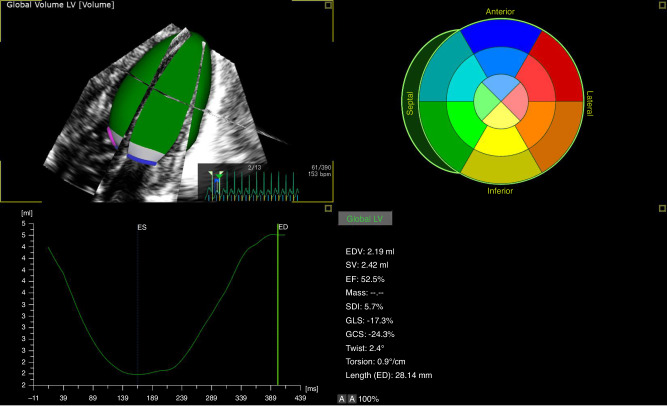
Offline analysis of three-dimensional echocardiographic left ventricular (LV) volume curve and function calculations. *ED* end-diastole, *ES* end-systole, *EDV* end-diastolic volume, *ESV* end-systolic volume, *SV* stroke volume, *EF* ejection fraction, *SDI* systolic dyssynchrony index, *GLS* global longitudinal strain, *GCS* global circumferential strain.

The LV endocardial border in the LV-focused four-chamber view was semi-automatically determined after two-point clicking of the LV apex and the center of the mitral valve annulus on the apical four-, two- and three-chamber views extracted from 3D echocardiography datasets. When required, the endocardial border was manually adjusted. The software generated time domain LV volume curves, and calculated the LV volume and LVEF (Fig. 1). LV end-diastolic volume (LVEDV), end-systolic volume (LVESV), stroke volume (LVSV) (calculated as the difference between LVEDV and LVESV), LVEF, global longitudinal strain (LVGLS), global circumferential strain (LVGCS), and torsion were automatically generated by the software (Fig. 1).

Three orthogonal planes and various landmarks in the apical RV-focused four-chamber view were selected to define the end-diastolic frames to obtain RV volume. According to the initial view adjustment, the program automatically supplied four chamber, sagittal, and coronal RV views, as well as RV end-diastolic volume (RVEDV), end-systolic volume (RVESV), stroke volume (RVSV) (calculated as the difference between RVEDV and RVESV), and ejection fraction (RVEF) ((Supplemental Fig. 1). Body size-dependent parameters were indexed by dividing by BW. LVCO and RVCO were calculated from the LVSV and RVSV, respectively, and heart rate at 3D volume measurements. Arterial elastance (Ea) was calculated as (0.9 × systolic blood pressure/stroke volume). End-systolic elastance (Ees) was calculated as (0.9 × systolic blood pressure / end-systolic volume (ESV).^1,33^ Echocardiographic Ea/Ees was also calculated to assess ventriculo-arterial coupling.^1,33^ We calculated Ea/Ees from ESV/ stroke volume of the LV and RV as measured by 3D echocardiography.

LA volume, LAEF, LA global longitudinal strain (LAGLS), and LA global circumferential strain (LAGCS) were calculated using the LV analysis software.

### Conventional transthoracic echocardiography

The following echocardiographic variables were measured: LV diastolic dimension (mm) and LV systolic dimension (mm) using the M-mode in the long-axis view; LA diameter (mm) and Ao diameter (mm) in the long-axis view using the leading edge method;^34^ LA area (cm^2^) and LA long-axis length (LA length, cm) in the four-chamber view; the narrowest internal diameter of the PDA (mm) using 2D echocardiography in the ductal long-axis view; the PDA flow pattern using the pulsed wave Doppler (left to right, right to left, bidirectional, none); and LPA end-diastolic velocity (edv) using the pulsed wave Doppler.^35–37^ RV function was evaluated by the fractional area change and corrected tricuspid annular plane systolic excursion (tricuspid annular plane systolic excursion/RV long diameter).^38^ We calculated LA volume using the single-plane area–length method in the four-chamber view with the following equation: LA volume = 0.85 × (LA area)^2^/(LA length) (cm^3^).^37^ The LV end-systolic wall stress was calculated using mean blood pressure measurements.^39,40^ Superior vena cava (SVC) flow were measured by pulsed wave Doppler as Kluckow and Evans reported.^41^ The mean of the maximum and minimum diameters measured from a still 2D image was determined over 5 heart cycles. The velocity time integral was calculated from the Doppler velocity tracings and averaged over 5 consecutive cardiac cycles. Heart rate was measured from the peak-to-peak intervals of the Doppler velocity time signals.

### Surgical technique

PDA ligation was performed employing standard methods under general anesthesia in intubated preterm infants using a standard technique, with intravenous infusions of fentanyl and the muscle relaxant pancuronium. A standard left posterolateral thoracotomy was performed through the third intercostal space with the patients placed in a right lateral position. A single ligation of the PDA was performed with a silk suture.

### Reproducibility analyses

Fifteen studies were randomly selected from the PDA group to investigate intraobserver variability, and one observer (K Toyoshima) measured LV and RV volumes at three month intervals. The observer at the second measurement was blinded to the results of the first measurement. A second observer (H Aoki), who was blinded to the results of the first observer, independently analyzed these data to investigate interobserver variability. Intraobserver and interobserver variabilities were examined using the intra-class correlation coefficient (ICC) and Bland–Altman analysis.

### Statistical analyses

Descriptive statistics (e.g., mean ± standard deviation, median [interquartile range]) were used to summarize the demographic or clinical data of preterm infants in the PDA and non-PDA groups. Differences between the two groups were analyzed using the unpaired t-test for continuous variables, Mann–Whitney U-test for median values, or Fisher’s exact test for categorical data. The hemodynamic, respiratory, and echocardiographic parameters were compared across the three time points using one-way analysis of variance with repeated measures.

Statistical analyses were performed with EZR (version 1.54) (Saitama Medical Center, Jichi Medical University, Saitama, Japan), which is a graphical user interface for R (The R Foundation for Statistical Computing, Vienna, Austria) and MedCalc (version 20; MedCalc Software Ltd., Ostend, Belgium, Belgium). A *P* value < 0.05 was considered significant.

## Results

### Clinical data of the PDA group

**Fig. 2:**
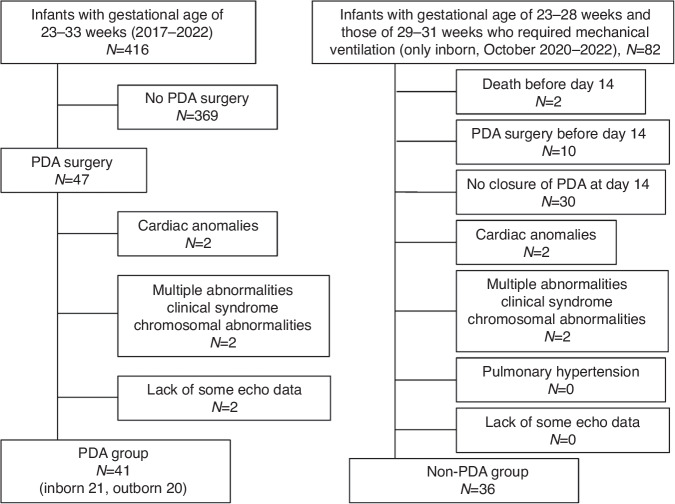
Study flow chart. **PDA group;** A total of 47 infants underwent PDA surgery with gestational age of 23–33 weeks (2017–2022) in our hospital. Six patients were excluded owing to the presence of cardiac anomalies (n = 2), multiple anomalies (n = 2), or the lack of some echocardiographic data (n = 2). Forty-one patients were included in the final analysis as PDA group, of whom 41 survived (100%). **Non-PDA group;** A total of 82 inborn infants with a gestational age between 23 and 28 weeks and those with a gestational age between 29 and 31 weeks who required mechanical ventilation were admitted to our hospital between October 2020 and 2022. Forty-Six patients were excluded owing to the death before day 14 (n = 2), PDA surgery before day 14 (n = 10), no closure of PDA at day 14 (n = 30), cardiac anomalies (n = 2), multiple anomalies (n = 2), Thirty-six patients were included in the final analysis as non-PDA group, of whom 35 survived (97.2%). *PDA* patent ductus arteriosus.

Forty-one preterm infants were enrolled in this study from 2017 to 2022 (Fig. 2). The median gestational age at birth and the median birth weight were 25 weeks (interquartile range [IQR]: 24–28) and 745 g (IQR: 592–940), respectively. Forty among 41 patients who underwent PDA surgery had undergone failed attempts at pharmacological closure with cyclooxygenase inhibitors. One preterm infant who underwent PDA surgery had progressive congestive heart failure where cyclooxygenase inhibitors were contraindicated (severe abdominal distension). The preterm infants underwent PDA surgery at 20 (IQR: 14–27) days of age at a median corrected gestational age of 29 weeks (IQR: 27–31) and had a median weight of 812 g (IQR: 643–970). The median length of the operation was 44 min (IQR: 38–48). The median fluid volume administered during PDA surgery was 27 ml/kg (IQR: 22–37). No critical complications were observed in any of the patients during the operation. Three-dimensional and conventional echocardiographic measurements were obtained in all preterm infants. There were no missing scans of the included patients. All 41(100%) preterm infants in this group (100%) survived to discharge.

### Reproducibility of 3D echocardiographic measurements

**Fig. 3:**
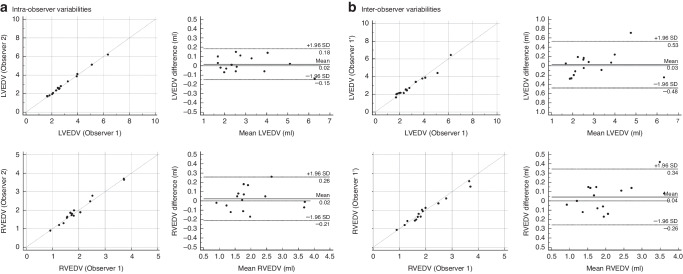
Intra- and interobserver variability assessment in ventricular volume. Intra- **a** and interobserver **b** variability assessment in ventricular volume. The three dashed lines show biases (means of differences) and limits of agreement. Bias is expressed as the mean of the difference (95% confidence interval). The limit of agreement is shown as the bias ± 2SDs. *LVEDV* left ventricular end-diastolic volume, *RVEDV* right ventricular end-diastolic volume, *SD* standard deviation.

**Fig. 4:**
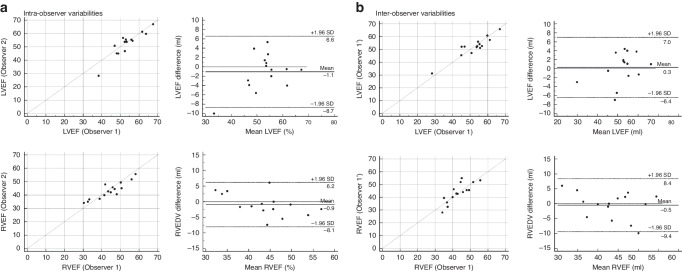
Intra- and interobserver variability assessment in ventricular contractility. Intra- **a** and interobserver **b** variability assessment in ventricular contractility. The three dashed lines show biases (means of differences) and limits of agreement. Bias is expressed as the mean of the difference (95% confidence interval). The limit of agreement is shown as the bias ± 2SDs. *LVEF* left ventricular ejection fraction, *RVEF* right ventricular ejection fraction, *SD* standard deviation.

The intra- and interobserver variability analysis in preterm infants with PDA, including the percentage bias, 95% limits of agreements, and ICCs for LVEDV, LVESV, LVSV, LVEF, RVEDV, RVESV RVSV, and RVEF, are shown in Table 1. Intrarater reproducibility (ICCs: LVEDV, 1.00; LVESV, 0.98; LVSV, 0.99; LVEF, 0.88; RVEDV, 0.99; RVESV, 0.98; RVSV, 0.97; and RVEF, 0.87) and interrater reproducibility (ICCs: LVEDV, 0.98; LVESV, 0.94; LVSV, 0.99; LVEF, 0.92; RVEDV, 0.98; RVESV, 0.97; RVSV, 0.94; and RVEF, 0.80) were excellent. Bland–Altman plots are shown in Figs. 3 and 4. The bias values were not significant, and the limits of agreement were acceptable.

**Table 1:** Intra- and interobserver variability of three-dimensional echocardiographic parameters in premature infants with patent ductus arteriosus.

	Bland–Altman plot		ICC (95% CI)
	Bias (95% CI)	95% LOA	
Intraobserver variability			
LVEDV	0.02 (−0.15 0.18)	−0.15 0.18	0.998 (0.994–0.999)
LVESV	0.05 (−0.02–0.11)	−0.18–0.28	0.983 (0.950–0.994)
LVSV	−0.02 (−0.07–0.02)	−0.19–0.14	0.993 (0.980–0.998)
LVEF	−1.05 (−3.21–1.10)	−8.69–6.58	0.883 (0.696–0.959)
RVEDV	0.02 (−0.05–0.09)	−0.21–0.26	0.989 (0.970–0.996)
RVESV	0.02 (−0.04–0.09)	−0.21–0.26	0.976 (0.932–0.992)
RVSV	0.00 (−0.04–0.04)	−0.15–0.14	0.972 (0.920–0.991)
RVEF	−0.93 (−2.94–1.09)	−8.05–6.20	0.874 (0.675–0.956)

	Bland–Altman plot		ICC (95% CI)
	Bias (95% CI)	95% LOA	
Interobserver variability			
LVEDV	0.03 (−0.12–0.17)	−0.48–0.53	0.981 (0.946–0.994)
LVESV	0.01 (−1.07–0.14)	−0.41–0.44	0.944 (0.843–0.981)
LVSV	0.01 (−0.06– 0.08)	−0.23–0.25	0.986 (0.959–0.995)
LVEF	0.27 (−1.62–2.17)	−6.43–6.98	0.920 (0.779–0.972)
RVEDV	0.04 (−0.04–0.13)	−0.26–0.34	0.980 (0.944–0.993)
RVESV	0.04 (−0.04–0.11)	−0.22–0.29	0.968 (0.910–0.989)
RVSV	0.01 (−0.05–0.07)	−0.20–0.21	0.941 (0.835–0.980)
RVEF	−0.50 (−3.00–2.01)	−9.38–8.38	0.800 (0.501–0.928)

ICC estimates and their 95% CIs were calculated using MedCalc® Statistical Software version 20 on the basis of a mean-rating (k = 2), absolute-agreement, two-way mixed-effects model.

*CI* confidence interval, *ICC* intraclass correlation coefficient, *LOA* limit of agreement,

*LVEDV* left ventricular end-diastolic volume, *LVESV* left ventricular end-systolic volume, *LVSV* left ventricular stroke volume, *LVEF*, left ventricular ejection fraction, *RVEDV* right ventricular end-diastolic volume, *RVESV* right ventricular end-systolic volume, *RVSV* right ventricular stroke volume, *RVEF* right ventricular ejection fraction.

### Comparison between the PDA and non-PDA groups

Figure 2 and Tables 2 and 3 show the comparison of demographic and echocardiographic data between preterm infants in the PDA group (*n* = 41) and those in the non-PDA group (n = 36). All patients with PDA had a left-to-right ductal shunt. All patients had left-to-right atrial shunt in both groups. There was no significant difference in gestational age, birth weight, sex, 1- and 5-min Apgar scores, or the small for gestational age rate between the two groups. There was also no significant difference in corrected gestational age, body weight, or heart rate on echocardiography between the two groups on echocardiography. The rate of in-hospital birth was lower in the PDA group than in the non-PDA group (P < 0.001). The median N-terminal prohormone brain natriuretic peptide concentration was significantly higher in the PDA group than in the non-PDA group (P < 0.001).

**Table 2:** Comparison of demographic and echocardiographic data between the PDA group and the non-PDA group at day 14.

	PDA	Non-PDA	*P* value
Number of patients	41	36	
Gestational age (weeks)	25 (24–28)	26.5 (25–28)	0.83
Birth weight (g)	745 (592–940)	849 (692–1045)	0.118
Male sex (%)	16 (39.0)	18 (50.0)	0.37
In-hospital birth (%)	21 (51.3)	34 (94.4)	< 0.001
Small for gestational age	8 (19.5)	7 (19.4)	1
Apgar score, 1 min	4 (2–6)	4 (3–6)	0.40
Apgar score, 5 min	7 (6–8)	7 (7–8)	0.63
Cyclooxygenase inhibitor (%)	40 (97.6)	15 (48.3)	< 0.001
Death (%)	0 (0)	1 (2.8)	0.47
Time of echocardiogram			
(days after birth)	20 (14–27)	14	
Corrected gestational age (weeks)	29 (27–31)	29 (27–30)	0.117
Body weight (g)	831 (643–970)	858 (645–1062)	0.86

Data are presented as the mean ± standard deviation, median (interquartile range), or number (%). Student’s t-test and Fisher’s exact test were used to analyze continuous and categorical variables, respectively. Differences in median values between the two groups were compared using the Mann–Whitney U-test.

*PDA* patent ductus arteriosus.

**Table 3:** Comparison of demographic and echocardiographic data between pre-and post-ligation in the PDA group, and between pre-ligation in the PDA group and the non-PDA group

		PDA				
Parameter	Non-PDA	Preoperatively	*P* value (vs non-PDA)	Postoperatively (4–8 h)	Postoperatively (24–48 h)	*P* value (3 point changes)
Time of echocardiogram after surgery (hours)				5 (4, 6)	29 (28, 31)	
Fluid volume (ml/kg/day)	156 (145–166)	120 (103–130)^a^	< 0.001	162 (142–176)^b^	142 (126–150)^bc^	< 0.001
Mean airway pressure (mmHg)	8 (8–10)	10 (8–12)	0.117	9 (8–10)	9 (8–10)	0.143
Respiratory severity score	1.9 (1.7–2.6)	2.2 (1.7–2.8)	0.57	2.1 (1.8–2.7)	2.3 (1.8–3.0)	0.29
Vasoactive–inotropic score	0 (0–0)	0 (0–0)	0.099	0 (0–0)	0 (0–0)	0.37
Systolic blood pressure (mm Hg) at echocardiogram	68.3 ± 11.3	57.1 ± 14.2^a^	< 0.001	50.7 ± 11.1	55.8 ± 9.0	0.068
diastolic blood pressure (mm Hg) at echocardiogram	37.1 ± 8.3	29.5 ± 8.5^a^	< 0.001	31.5 ± 7.4	32.3 ± 5.9	0.013
Heart rate (beats/min) at echocardiogram	156 ± 12	153 ± 15	0.28	145 ± 17^b^	143 ± 12^b^	< 0.001
NT-proBNP (pg/ml)	772 (505–1460)	12266 (8030–25478)^a^	< 0.001		3695 (2299–6029)^b^	< 0.001
PDA diameter (mm)	0.0 ± 0.0	2.1 ± 0.8^a^	< 0.001			
LPA EDV (cm/s)	8.9 ± 1.9	25.3 ± 9.9^a^	< 0.001	6.5 ± 4.3^b^	7.4 ± 3.0^b^	< 0.001
LVDD (mm)	11.8 ± 1.6	14.7 ± 2.3^a^	< 0.001	11.8 ± 2.3^b^	12.7 ± 1.8^b,c^	< 0.001
LVEF (M mode) (%)	64.2 ± 6.7	70.9 ± 7.9^a^	< 0.001	59.0 ± 8.7^b^	58.7 ± 9.0^b^	< 0.001
ESWS (g/cm^2^)	33.3 ± 9.6	36.3 ± 16.4^a^	0.33	26.9 ± 12.4^b^	36.2 ± 13.4^c^	< 0.001
LA/Ao	1.10 ± 0.16	1.63 ± 0.17^a^	< 0.001	1.12 ± 0.16^b^	1.23 ± 0.17^b,c^	< 0.001
LAVI (ml/kg)	0.71 ± 0.23	1.73 ± 0.49^a^	< 0.001	0.82 ± 0.27^b^	0.91 ± 0.27^b^	< 0.001
LAEF (%)	56.4 ± 10.7	50.0 ± 14.3^a^	0.009	49.9 ± 10.6	53.2 ± 10.7	0.24
RVFAC	35.5 ± 9.8	22.3 ± 9.8^a^	< 0.001	30.5 ± 10.8^b^	35.2 ± 11.4^b^	< 0.001
cTAPSE (%)	38.0 ± 5.5	34.0 ± 7.5^a^	0.01	29.2 ± 7.9^b^	33.6 ± 5.8^c^	0.001
SVCF (ml/kg/min)	191 ± 77	213 ± 80	0.27	157 ± 60^b^	172 ± 39	0.007
3D echocardiography						
LVEDV (ml)	1.91 ± 0.56	3.21 ± 1.23^a^	< 0.001	2.00 ± 0.87^b^	2.43 ± 0.89^b^	< 0.001
LVEDV/BW (ml/kg)	2.30 ± 0.47	3.80 ± 0.68^a^	< 0.001	2.38 ± 0.65^b^	2.93 ± 0.65^b,c^	< 0.001
LVESV (ml)	1.06 ± 0.33	1.51 ± 0.61^a^	< 0.001	1.23 ± 0.49^b^	1.47 ± 0.57^c^	< 0.001
LVESV/BW (ml/kg)	1.27 ± 0.26	1.79 ± 0.41^a^	< 0.001	1.48 ± 0.43^b^	1.79 ± 0.60’^c^	< 0.001
LVSV (ml)	0.85 ± 0.30	1.70 ± 0.69^a^	< 0.001	0.77 ± 0.43^b^	0.98 ± 0.43^b,c^	< 0.001
LVSV/BW (ml/kg)	1.03 ± 0.28	2.01 ± 0.44^a^	< 0.001	0.90 ± 0.3^b^	1.16 ± 0.28^b^	< 0.001
LVEF (%)	43.8 ± 7.3	53.0 ± 6.7^a^	< 0.001	37.5 ± 9.3^b^	40.1 ± 8.1^b^	< 0.001
LVCO (ml/kg/min)	159 ± 46	307 ± 75^a^	< 0.001	131 ± 55^b^	166 ± 44^b,c^	< 0.001
LVGLS (%)	−15.3 ± 2.7	−20.8 ± 3.0^a^	< 0.001	−12.1 ± 4.1^b^	−14.7 ± 3.3^b^	< 0.001
LVGCS (%)	−19.2 ± 4.4	−21.6 ± 8.9	0.136	−15.7 ± 4.9^b^	−16.5 ± 4.5^b^	< 0.001
LV torsion	2.73 ± 1.81	3.60 ± 2.38	0.078	2.04 ± 1.76	3.64 ± 4.04	0.052
LV Ea (mmHg/ml/kg)	64.9 ± 21.8	26.0 ± 9.5^a^	< 0.001	59.7 ± 31.7^b^	46.1 ± 14.5^b,c^	< 0.001
LV Ees (mmHg/ml/kg)	49.9 ± 10.8	29.1 ± 9.4^a^	< 0.001	32.6 ± 8.9	30.1 ± 8.2	0.019
LV Ea/Ees	1.31 ± 0.37	0.92 ± 0.25^a^	< 0.001	1.89 ± 1.03^b^	1.65 ± 0.88^b^	< 0.001
RVEDV (ml)	1.87 ± 0.47	2.37 ± 1.14^a^	0.017	1.81 ± 0.82^b^	2.12 ± 0.76^c^	< 0.001
RVEDV/BW (ml/kg)	2.29 ± 0.44	2.80 ± 0.86^a^	0.001	2.18 ± 0.86^b^	2.54 ± 0.53^c^	< 0.001
RVESV (ml)	0.91 ± 0.29	1.26 ± 0.66^a^	< 0.001	1.02 ± 0.54^b^	1.08 ± 0.43^b^	< 0.001
RVESV/BW (ml/kg)	1.12 ± 0.30	1.49 ± 0.53^a^	< 0.001	1.22 ± 0.45^b^	1.30 ± 0.37^b^	< 0.001
RVSV (ml)	0.96 ± 0.25	1.11 ± 0.54	0.135	0.79 ± 0.43^b^	1.04 ± 0.40^c^	< 0.001
RVSV/BW (ml/kg)	1.17 ± 0.24	1.32 ± 0.43	0.083	0.96 ± 0.43^b^	1.24 ± 0.29^c^	< 0.001
RVEF (%)	51.5 ± 6.6	47.2 ± 7.5^a^	0.010	43.9 ± 11.6	48.9 ± 7.5	0.076
RVCO (ml/kg/min)	182 ± 37	201 ± 68	0.099	140 ± 66^b^	178 ± 42^c^	< 0.001
RV Ea/Ees	0.98 ± 0.28	1.17 ± 0.34^a^	0.008	1.49 ± 0.80	1.09 ± 0.35^c^	0.019
LVEDV/RVEDV ratio	1.02 ± 0.20	1.45 ± 0.38^a^	< 0.001	1.17 ± 0.39^b^	1.18 ± 0.28^b^	< 0.001
LVSV/RVSV ratio	0.89 ± 0.24	1.69 ± 0.64^a^	< 0.001	1.08 ± 0.57^b^	0.96 ± 0.23^b^	< 0.001
LA maximum volume (ml)	0.54 ± 0.20	1.37 ± 0.58^a^	< 0.001	0.71 ± 0.34^b^	0.81 ± 0.39^b^	< 0.001
LA maximum volume/BW (ml/kg)	0.65 ± 0.21	1.64 ± 0.45^a^	< 0.001	0.84 ± 0.28^b^	0.96 ± 0.30^b^	< 0.001
LA minimum volume (ml)	0.29 ± 0.11	0.79 ± 0.32^a^	< 0.001	0.42 ± 0.19^b^	0.45 ± 0.24^b^	< 0.001
LA minimum volume/BW (ml/kg)	0.35 ± 0.13	0.97 ± 0.29^a^	< 0.001	0.50 ± 0.17^b^	0.55 ± 0.22^b^	< 0.001
LAEF (%)	46.4 ± 8.3	41.0 ± 9.3^a^	0.009	38.6 ± 11.1	45.0 ± 10.4^c^	0.012
LAGLS (3D) (%)	17.6 ± 8.8	18.3 ± 5.7	0.67	15.7 ± 6.5	19.9 ± 6.6	0.003
LAGCS (3D) (%)	14.5 ± 6.4	11.3 ± 4.9^a^	0.016	13.3 ± 6.7	15.2 ± 4.7^b^	0.014

Differences between two groups were analyzed using the unpaired t-test for continuous variables, the Mann–Whitney U-test for median values, and Fisher’s exact test for categorical data. Hemodynamic, respiratory, and echocardiographic parameters were compared across the three time points using one-way analysis of variance with repeated measures.

^a^*P* < 0.05, the non-PDA group vs preoperatively in the PDA group;

^b^*P* < 0.05, vs preoperatively;

^c^*P* < 0.05, vs 4–8 h after surgery.

*PDA* patent ductus arteriosus, *NT-proBNP* N-terminal pro-brain natriuretic peptide, *2D* two-dimensional, *LPA* left pulmonary artery, *EDV* end-diastolic velocity, *LVDD* left ventricular diastolic dimension, *LVEF* left ventricular ejection fraction, *ESWS* end-systolic wall stress, *LA/Ao* left atrial diameter to aortic diameter ratio, *LAVI* left atrial volume index, *LAEF* left atrial emptying fraction, *RVFAC* right ventricular fractional area change, c*TAPSE* corrected tricuspid annular plane systolic excursion, *SVCF* doppler volumetric measurements of superior vena cava flow, *3D* three-dimensional, *LVEDV* left ventricular end-diastolic volume, *LVESV* left ventricular end-systolic volume, *LVSV* left ventricular stroke volume, *LVCO* left ventricular cardiac output, *LVGLS* left ventricular global longitudinal strain, *LVGCS* left ventricular global circumferential strain, *LV torsion* left ventricular torsion, *LVEa/Ees* left ventricular arterial elastance to end-systolic elastance ratio, *RVEDV* right ventricular end-diastolic volume, *RVESV* right ventricular end-systolic volume, *RVSV* right ventricular stroke volume, *RVEF* right ventricular ejection fraction, *RVCO* right ventricular cardiac output, *RVEa/Ees* right ventricular arterial elastance to end-systolic elastance ratio, *LA maximum volume* left atrial maximum volume, *LA minimum volume* left atrial minimum volume, *LAGLS* left atrial global longitudinal strain, *LAGCS* left atrial global circumferential strain, *BW* body weight.

**Fig. 5:**
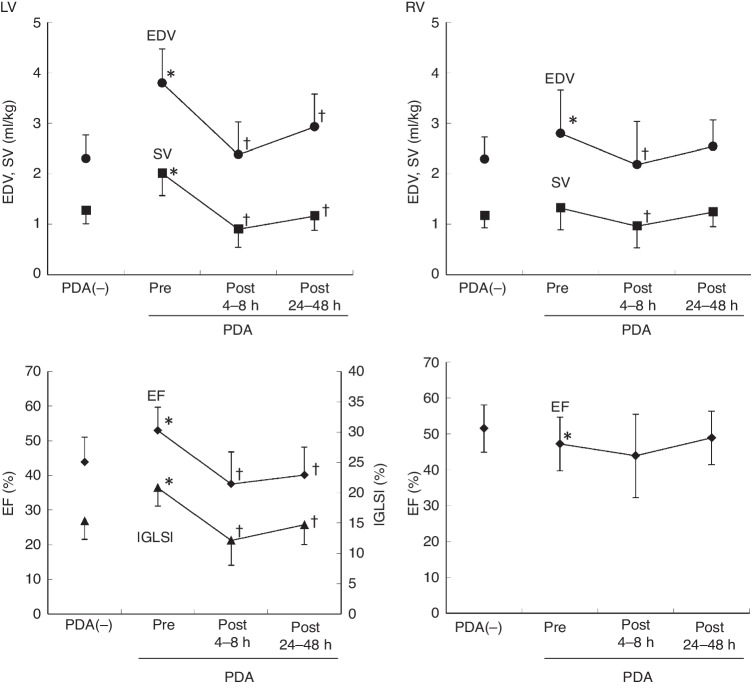
Comparison among non-PDA, pre-ligation, post-ligation at 4–8 h, and post-ligation at 24–48 h. EDV/body weight (black circles), SV/body weight (black squares), EF (black diamonds) and LVGLS (black triangles) are shown in the non-PDA and PDA groups. Preoperatively, the LV had an increased EDV, EF, GLS, and SV compared with those of non-PDA. At 4–8 h postoperatively, the LV had a decreased EDV, EF, GLS, and SV compared with those at pre-ligation. At 24–48 h postoperatively, LV contractility remained subnormal, but SV was increased owing to the increased EDV. Preoperatively, the RV had an increased EDV and reduced EF, and comparable SV compared with those of non-PDA. At 4–8 h postoperatively, the RV had a reduced SV due to a reduced EDV and persisting RV dysfunction compared with those at pre-ligation. At 24–48 h postoperatively, the RV showed recovery of SV owing to recovered EDV and RV function. Each plot and error bar represent the mean and standard deviation, respectively. PDA(-), non-PDA. *P < 0.05, versus non-PDA. †*P* < 0.05, versus at pre-ligation. *LV* left ventricle, *RV* right ventricle, *EDV* end-diastolic volume, *SV* stroke volume, *EF* ejection fraction, *GLS* global longitudinal strain, *PDA* patent ductus arteriosus.

Blood pressure was measured using the arterial line in 27/41 (66%) preterm infants at pre-ligation. Blood pressure was measured with the oscillometer technique in 14/41 (34%) preterm infants at pre-ligation in the PDA group and in all preterm infants in the non-PDA group. Systolic and diastolic blood pressure was lower in the PDA group than in the non-PDA group (both P < 0.001). Not only LV volume (LVEDV/BW and LVESV/BW) but also RV volume (RVEDV/BW and RVESV/BW) measured by 3D echocardiography before surgery in the PDA group were significantly larger than those in the non-PDA group (Table 3 and Fig. 5). There was increased LV wall motion in the PDA group as shown by a significantly larger LVEF, LVGLS, and LVGCS in the PDA group than in the non-PDA group (Table 3 and Fig. 5). In contrast, decreased RV wall motion was indicated by a significantly smaller RVEF and corrected tricuspid annular plane systolic excursion in the PDA group than in the non-PDA group (Table 3).

**Fig. 6:**
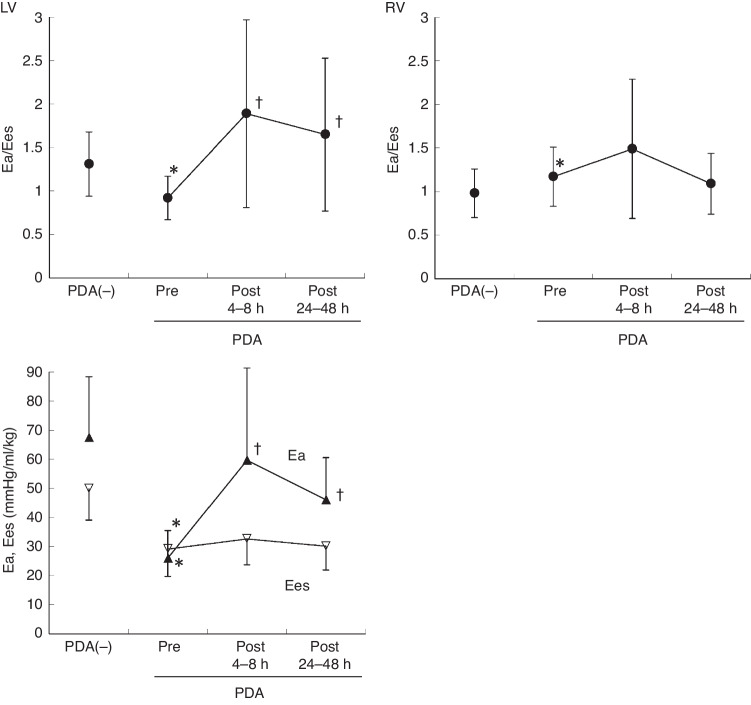
Comparison of left and right ventricular Ea/Ees among no PDA, pre-ligation, post-ligation at 4–8 h, and post-ligation at 24–48 h. The Ea/Ees (black circles), LV Ea (black triangles), and LV Ees (white triangles) are shown in the non-PDA and PDA groups. Preoperatively, the LV had a lower Ea/Ees because of a lower Ees and even lower Ea. At 4–8 h postoperatively, Ea/Ees was significantly increased owing to a significant increase in Ea and unchanged Ees. At 24–48 h postoperatively, Ea/Ees showed a tendency for improvement because of decreased Ea and unchanged Ees. Preoperatively, the RV had an increased Ea/Ees. RV Ea/Ees showed a further increase at 4–8 h postoperatively. At 24–48 h postoperatively, RV Ea/Ees showed an improvement. Each plot and error bar represent the mean and standard deviation, respectively. PDA(-), non-PDA. *P < 0.05, versus at pre-ligation. *LV* left ventricle, *RV* right ventricle, *Ea* arterial elastance, *Ees* end-systolic elastance.

The LV Ea was significantly lower in the PDA group than in the non-PDA group (*P* < 0.001) (Table 3 and Fig. 6). The LV Ea/Ees ratio in the PDA ligation group was significantly lower than that in the non-PDA group (P < 0.001). However, the RV Ea/Ees ratio was larger in the PDA group than in the non-PDA group (*P* < 0.001) (Table 3 and Fig. 6).

The indices of LA enlargement (LA/Ao, LA volume) were higher in the PDA group than in the non-PDA group (P < 0.001). The LAEF in 2D and 3D echocardiography and LAGCS was lower in the PDA group than in the non-PDA group (*P* < 0.01), but there was no significant difference in LAGLS.

There was no significant difference in RVCO or SVC flow between the two groups. However, LVCO in the PDA group was 1.9-fold larger than that in the non-PDA group (*P* < 0.001).

### Comparison among pre-ligation, post-ligation at 4–8 h, and post–ligation at 24–48 h

Serial hemodynamics, respiratory data, and echocardiographic parameters are shown in Table 3. Blood pressure was measured using the arterial line in 27/41 (66%) preterm infants at pre-ligation and in all preterm infants at post-ligation. Blood pressure was measured with the oscillometer technique in 14/41 (34%) infants at pre-ligation. The heart rate at 4–8 and 24–48 h postoperatively was significantly lower than that preoperatively (both *P* < 0.05).

The LVDD was significantly lower at 4–8 and 24–48 h postoperatively than preoperatively (both *P* < 0.001). The left pulmonary artery end-diastolic velocity, LVEF, LA/Ao ratio, LA volume index, and SVC flow in 2D echocardiography were significantly lower postoperatively than preoperatively (*P* < 0.01). Three-dimensional LV and LA volume parameters (LVEDV/BW, LVESV/BW, RVEDV/BW, RVESV/BW, LA maximum volume/BW and LA minimum volume/BW) were significantly lower at 4–8 h postoperatively than preoperatively. LVEDV/BW was significantly decreased at 4–8 h postoperatively compared with preoperatively (*P* < 0.001), and then increased at 24–48 h postoperatively (*P* < 0.001) (Fig. 5). LV 3D contraction parameters (LVEF, LVGLS, and LVGCS) were reduced at 4–8 h and remained unrecovered at 24–48 h postoperatively. The LVEF (3D) was significantly decreased at 4–8 h postoperatively compared with preoperatively (*P* < 0.001), and it did not change at 24–48 h postoperatively (Fig. 5). LVGLS (3D) was significantly decreased at 4–8 h postoperatively compared with preoperatively (P < 0.001), and was slightly increased at 24–48 h postoperatively (*P* < 0.01).

LV Ea (3D) was significantly increased at 4–8 h postoperatively compared with preoperatively (*P* < 0.001), and was decreased at 24–48 h postoperatively (*P* < 0.01) (Fig. 6). LV Ea/Ees (3D) was significantly increased at 4–8 h postoperatively compared with preoperatively (P < 0.001), and did not change at 24–48 h postoperatively (Fig. 6). LVCO (3D) was significantly decreased at 4–8 h postoperatively compared with preoperatively (*P* < 0.001), and was increased at 24–48 h postoperatively (*P* < 0.01).

RVEDV/BW was significantly decreased at 4–8 h postoperatively compared with preoperatively (*P* < 0.001), and was increased at 24–48 h postoperatively (*P* < 0.001) (Fig. 5). RVCO (3D) was significantly decreased at 4–8 h postoperatively compared with preoperatively (*P* < 0.001), and was increased at 24–48 h postoperatively (*P* < 0.01). There was no significant change in the RVEF among the different time points (*P* = 0.08) (Fig. 5). RV Ea/Ees (3D) was increased at 4–8 h, but was then decreased at 24–48 h postoperatively compared with preoperatively (*P* = 0.02) (Fig. 6).

LA maximum volume was significantly decreased at 4–8 h postoperatively compared with preoperatively (*P* < 0.001), and did not change at 24–48 h postoperatively.

## Discussion

In this study, we assessed LV, RV, and LA loading conditions and function in preterm infants who underwent surgical closure of PDA. We showed the reliability of 3D echocardiographic parameters in serial evaluations before and after PDA ligation. Although PDA is believed to be a left heart disease, this study showed the following. (1) Preterm PDA involved loading and functional abnormalities in the left and right heart. (2) PDA ligation significantly reduced not only LV and LA volumes, but also the RV volume, and ventriculo-arterial coupling was impaired with a reduced output at 4–8 h after the operation. (3) The CO was increased at 24–48 h after surgery owing to the recovered loading condition, although LV systolic dysfunction remained. Understanding these changes may contribute to the optimal individual management of preterm infants complicated by PDA.

### Comparison between preterm infants with PDA and those without PDA

This study demonstrated a successful quantification of the LV, RV, and LA volumes by 3D echocardiography in preterm infants with PDA and in those without PDA. The LV and RV volumes corrected by BW in preterm infants in the non-PDA group are consistent with those reported in previous studies that evaluated healthy newborns.^24,25^ The LVEDV/BW, RVEDV/BW, and LA maximum volume/BW in the PDA group were 160%, 127%, and 195% of that in the non-PDA group, respectively.

The 3D analysis employed in this study is semi-automatic and traces the LV, RV, or LA endocardial border. This type of analysis can directly calculate the EF from the LV and RV volume curves using speckle tracking without using geometric assumption. We observed that preterm infants with a large PDA preoperatively had a higher LVEF and lower RVEF than preterm infants without PDA. Physiological insights on LV adaptation before and after PDA closure can be gained by an assessment of ventriculo-arterial coupling.^8,42,43^ Ventriculo-arterial coupling refers to the effect of the load imposed by the arterial system (Ea) on ventricular systolic performance (Ees)^44–46^ and is closely related to cardiac energetics.^47^ Nagata et al. reported the changes in ventriculo-arterial coupling in a cohort of preterm infants who underwent surgical ligation and observed that ventricular efficiency transiently deteriorated in the first 24 h after PDA surgical closure, accompanied by an increase in Ea and in Ea/Ees.^8^ Gray et al. demonstrated a pre-procedural Ea/Ees threshold to predict the need for postoperative vasoactive support.^42^ Bischoff et al. reported that 17 (48.6%) patients developed the hemodynamic instability after PDA surgical closure, which was associated with a younger age, lower preload, and higher Ea and Ees.^43^ Three-dimensional echocardiography can directly measure EDV and ESV, which allows an evaluation of ventriculo-arterial coupling by Ea/Ees in both ventricles. In this study, the preoperative LV Ea/Ees ratio in the PDA group was smaller than that in the non-PDA group (Fig. 6). This finding reflects the reduced LV afterload against LV contractility due to the left-right shunt across the PDA at the cost of left heart volume overload, increased pulmonary flow, and steal of systemic organ flow.

RV volume and the RVEF cannot be assessed by conventional 2D echocardiography. To our knowledge, this is the first study using 3D echocardiography to demonstrate that that RVEDV is larger and the RVEF is lower preoperatively in preterm infants with PDA than in those without PDA. Preoperatively the RV had impaired ventriculo-arterial coupling as shown by an increased Ea/Ees ratio (Fig. 6). These results indicate that preterm infants with PDA requiring surgery have RV functional abnormality, even though PDA is traditionally believed to be left heart disease. These observations may be attributed to several factors, such as increased RV preload due to a left-to-right shunt via the foramen ovale, increased RV afterload due to elevated left atrial and pulmonary arterial pressure, and decreased coronary flow by increased PDA flow.

### Effect of PDA ligation on hemodynamics

In this study, LV, RV, and LA volumes and contractility were reduced with impaired ventriculo-arterial coupling at 4–8 h after PDA ligation, and these tended to recover at 24–48 h after surgery. LV, RV and LA volumes were acutely reduced immediately after PDA ligation. The greater volumes in the preoperative state likely reflect an increased LV and RV preload due to PDA and PFO shunt. After PDA ligation, ventricular volumes returned to within the normal range. The LVEF was markedly decreased with a normalized LV preload, and resulted in a lower LV output at 4–8 h after PDA ligation. Although the LVEF remained low, LVCO recovered owing to the recovered LVEDV at 24–48 h after PDA ligation. These observations are consistent with a previous finding that increased CO in preterm infants with PDA largely depends on the Frank–Starling relationship and not on changes in contractility and heart rate.^6,48,49^ This finding indicated that acute unloading may considerably reduce CO in the chronically volume-overloaded LV in preterm infants. At 4–8 h postoperatively in the PDA group, the LV had a lower EF under lower blood pressure and LVEDV was decreased, but it was similar to the non-PDA group. The heart rate was decreased. These data indicated the following: (1) the LV had systolic dysfunction that required a greater LVEDV, but (2) LVEDV was decreased to a normal volume and stroke volume was decreased, and (3) the reduction in stroke volume and heart rate resulted in the reduction in LVCO.

Preoperatively, patients with PDA had a much lower LV afterload status preoperatively (Ea, Fig. 6) than those without PDA owing to chronic exposure of the LV to low pulmonary vascular resistance through the ductus. PDA closure resulted in transient deconditioning of the LV and afterload mismatch owing to an abrupt increase in the LV afterload (Ea) and lack of change in LV contractility as shown by no significant change in Ees (Fig. 6). Preoperative chronic coronary steal may contribute to this deconditioning. The effects of anesthesia may also play an important role in this condition because of the reduction in heart rate.

In this study, RV volume was reduced, although not as much as that of LV volume, with a reduced RVEF and impaired ventriculo-arterial coupling. This situation resulted in a reduced output at 4–8 h after the operation. These changes in the RV were almost recovered at 24–48 h after PDA surgery. The initial reduction in RV volume could be partly explained by a reduced atrial left-to-right shunt, decreased systemic venous return by the decreased LV output, and a reduction in RV afterload after PDA ligation.

### Study limitations

This study has several limitations. First, because none of the included patients developed post-ligation instability syndrome, and because the sample size of this study was small, we could not assess the effect of 3D compared with 2D echocardiographic evaluation on the clinical outcome. However, this study showed serial hemodynamic detail not only subacutely (24–48 h), but also acutely (4–8 h), after PDA ligation in preterm infants using 3D echocardiography, which does not use geometric assumption to measure ventricular volume. Second, this was a single-center study. There are considerable differences in the evaluation and management of preterm infants with PDA between centers and between countries. Postoperative changes are highly variable depending on the preoperative conditions, management, and intraoperative anesthesia. Therefore, the external validity of the present results needs to be carefully considered.

Blood pressure was measured using an arterial line at all post-operative time points in PDA cases, but it was measured with the oscillometer technique in all non-PDA cases and only in 14/41(34%) preoperatively. The method of blood pressure measurement does not affect systolic and mean blood pressure as much as diastolic blood pressure.^50^ However, interpretation of blood pressure and blood pressure-based variables should take into account the difference in methods of measuring blood pressure.

The echocardiographer was not blinded to the clinical information or background data of the included patients at the 3D data extraction. However, the data extraction processes were semiautomatic, and the intraobserver and interobserver variability was acceptable. We used only one vendor’s algorithm. Finally, Ees was calculated using the assumption that the volume intercept of the end–systolic pressure–volume relationship (ESPVR) is equals to 0 (ml).^1,33^ Although this method has a limitations, we used it because it has often been used in clinical studies^1,33^ to assess loading conditions and cardiac function separately and in an integrated manner. Inducing changes in the loading condition to generate multiple pressure–volume relationships in preterm infants may be impossible or inappropriate. Additionally, to date, there has been no single beat estimation of Ees has been validated in this population.

## Conclusions

Hemodynamic abnormalities in PDA and its postoperative status involve the left and right heart, and 3D echocardiographic evaluation is reliable for improving the understanding of these abnormalities in preterm infants. A future prospective study is warranted to clarify whether the assessment of LV and RV volume, contractility, and ventriculo-arterial coupling with 3D echocardiography may help optimize individual management to improve the prognosis in this population.

## Supplementary Information

Supplemental Figure 1

Supplemental Figure Legend

## Data Availability

The data supporting the results of this study are available on reasonable request to the corresponding author (K Toyoshima).
