# Supplementary figures and images for: Biventricular function in preterm infants with patent ductus arteriosus ligation: A three-dimensional echocardiographic study
Source: Pediatr Res. 2024 Apr 13;96(3):773–84. doi: 10.1038/s41390-024-03180-w (PMC11499271; doi:10.1038/s41390-024-03180-w)

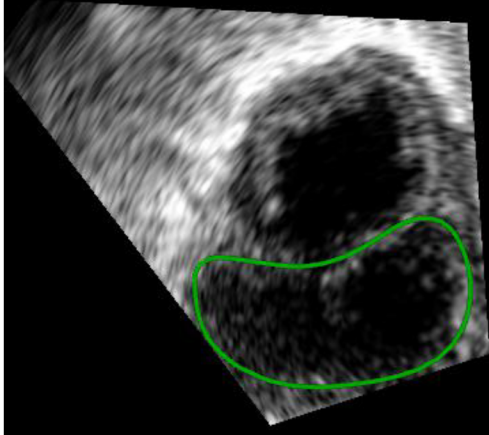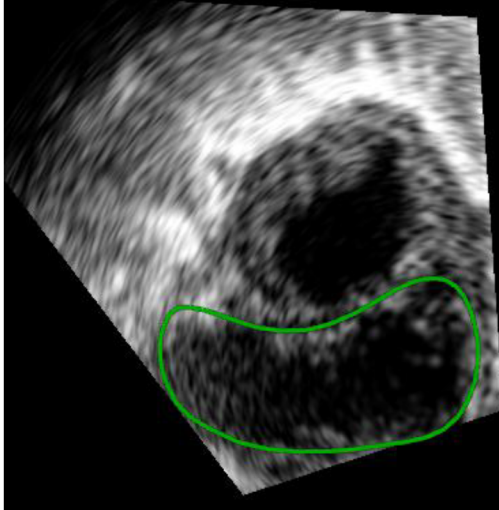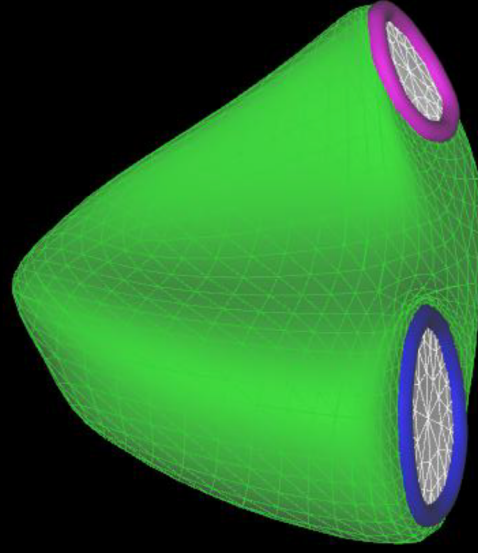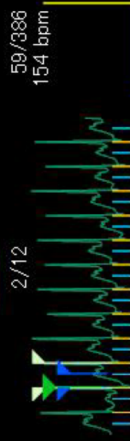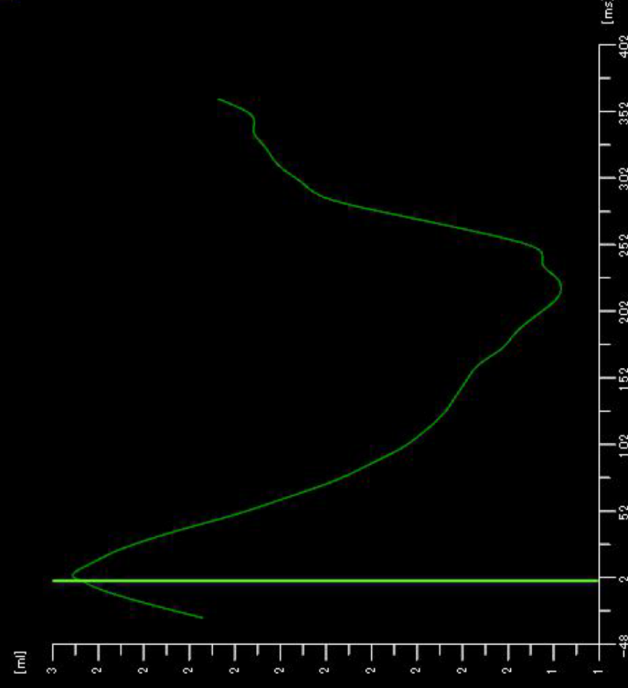

EDV: 2.46 ml  
ESV: 1.38 ml  
SV: 1.07 ml  
EF: 43.7 %

Supplement: Supplementary file 1 — Supplemental Figure 1 [file 41390_2024_3180_MOESM1_ESM.pdf]
