# Supplementary material for: Biventricular function in preterm infants with patent ductus arteriosus ligation: A three-dimensional echocardiographic study
Source: Pediatr Res. 2024 Apr 13;96(3):773–84. doi: 10.1038/s41390-024-03180-w (PMC11499271; doi:10.1038/s41390-024-03180-w)
Supplement: Supplementary file 2 — Supplemental Figure Legend [file 41390_2024_3180_MOESM2_ESM.pdf]

Supplemental Figure Legend

Supplemental Fig. 1 Offline analysis of three-dimensional echocardiographic right ventricular (RV) volume curve and function calculations. The green lines indicate the semi-automatically detected RV internal border.

4Ch four chamber, *SAX* short axis, *EDV* end-diastolic volume, *ESV* end-systolic volume, *SV* stroke volume, EF, ejection fraction.
